# Morphology, ultrastructure, and molecular phylogeny of the ciliate *Sonderia vorax* with insights into the systematics of order Plagiopylida

**DOI:** 10.1186/1471-2180-13-40

**Published:** 2013-02-18

**Authors:** Letizia Modeo, Sergei I Fokin, Vittorio Boscaro, Ilaria Andreoli, Filippo Ferrantini, Giovanna Rosati, Franco Verni, Giulio Petroni

**Affiliations:** 1 Unit of Protistology-Zoology, Department of Biology, University of Pisa, via A. Volta 4/6, Pisa 56126, Italy; 2 Department of Invertebrate Zoology, St. Petersburg State University, Universitetskaya emb. 7/9, St. Petersburg, 199034, Russia

**Keywords:** Ectosymbionts, FISH, Hydrogenosomes-endosymbionts assemblages, Oxygen deficient environment, Plagiopylida, Phylogeny, Silver-nitrate staining, SSU rRNA, Sulphide fauna, Ultrastructure

## Abstract

**Background:**

Ciliates of the family Sonderiidae are common members of the eukaryotic communities in various anoxic environments. They host both ecto- and endosymbiotic prokaryotes (the latter associated with hydrogenosomes) and possess peculiar morpho-ultrastructural features, whose functions and homologies are not known. Their phylogenetic relationships with other ciliates are not completely resolved and the available literature, especially concerning electron microscopy and molecular studies, is quite scarce.

**Results:**

*Sonderia vorax* Kahl, 1928 is redescribed from an oxygen-deficient, brackish-water pond along the Ligurian Sea coastlines of Italy. Data on morphology, morphometry, and ultrastructure are reported. *S. vorax* is ovoid-ellipsoid in shape, dorsoventrally flattened, 130 x 69 μm (mean *in vivo*); it shows an almost spherical macronucleus, and one relatively large micronucleus. The ventral kinetom has a “secant system” including fronto-ventral and fronto-lateral kineties. A distinctive layer of bacteria laying between kineties covers the ciliate surface. Two types of extrusomes and hydrogenosomes-endosymbiotic bacteria assemblages are present in the cytoplasm. The phylogeny based on 18S rRNA gene sequences places *S. vorax* among Plagiopylida; Sonderiidae clusters with Plagiopylidae, although lower-level relationships remain uncertain. The studied population is fixed as neotype and the ciliate is established as type species of the genus, currently lacking.

**Conclusions:**

This is the first description of a representative of Sonderiidae performed with both morphological and molecular data. To sum up, many previous hypotheses on this interesting, poorly known taxon are confirmed but confusion and contradictory data are as well highlighted.

## Background

The genus *Sonderia* was established by Kahl in 1928 [1] for ciliates collected from Oldesloe salt marshes (Hamburg region, Germany) and later on from the Island of Sylt (North Sea, Germany). These interesting ciliates are ubiquitous and common in the sapropelic environment of salt marshes and in ecologically similar brackish water sites with oxygen deficiency [2-9].

Within the genus *Sonderia* quite conspicuous, ovoid-ellipsoid ciliates are included. These are dorsoventrally flattened, uniformly ciliated, and show a conical, subapical oral cavity. The cell length of genus representatives varies, according to the species, from 80 to 250 μm. Their surface is covered by a distinctive layer of bacteria organized in parallel along the host body axis and plunged in a gelatinous coating between kineties. From a few to numerous long, needle-shaped extrusomes are present in the cortex. Oral kineties extend from somatic ones, both organized as monokinetids, and run perpendicularly to the upper and lower lips of the oral cavity opening. A transversely striated band arises near the right margin of the oral cleft and dorsolaterally runs down [10-13].

After Kahl, 1928 [1] the genus *Sonderia* was splitted into four related genera – *Sonderia, Parasonderia, Kahlisonderia,* and *Oncosonderia*[14-16]. All these genera share some morphological features such as: general shape, subapical position of oral cavity, transversely striated band passing from the oral cleft, and a surface gelatinous layer with embedded bacteria. However, silver impregnation and/or different staining methods were never applied to describe the majority of the species and the type species of *Sonderia* was never established [15,17]. As a curiosity, six out of eight species described during the genus establishment were marked in the key publication [11] as “Kahl, 1930”, but no articles dealing with *Sonderia* published in 1930 are in fact available [15,18].

Molecular studies on *Sonderia* lack at present, while there is a single study on a representative of the family Sonderiidae, *Parasonderia vestita*[16]. Only a few 18S rRNA gene sequences are available for other taxa belonging to the class Plagiopylea, namely the plagiopylids *Plagiopyla*[19,20], *Lechriopyla*[21] and *Trimyema*[19,22-24], and the odontostomatid *Epalxella*[25]. The fact that these sequences form a clade is the main uniting feature of the class itself [12]. Members of the class Prostomatea usually appear to be the most closely related to Plagiopylea in phylogenetic analyses.

In the present paper *Sonderia vorax* Kahl, 1928 is redescribed as type species of the genus and neotypified using a modern multidisciplinary analytical approach which combines morphological (i.e. live, stained, scanning, and transmission electron microscopy) with morphometric and molecular analysis.

## Methods

### General remarks

The neotype population of *Sonderia vorax* was discovered in three brackish water samples with a 4–8% salinity range; together with oxygen level (see below), salinity was measured using an OX 22 oxygen meter (Aqualytic, Langen, Germany). All the samples came from the same brackish water pond referred to as “Stagno 1” placed on the coastline of Ligurian Sea, close to Serchio River mouth, Pisa district (Tuscany, Italy) (43°47’39” N, 10°16’4” E), and were collected during October 2005, with a water temperature ranging from 18 to 22°C. The samples also contained ciliates such as *Sonderia pharyngea*, *Plagiopyla* sp., *Copemetopus* sp., and *Metopus* sp. in moderate or low abundance. In the sediment layer, where *S. vorax* was mainly discovered, the oxygen level in water was 1–7%; close to the water surface it was 35–66%. Attempts to cultivate in laboratory *S. vorax* were unsuccessful under full oxygen conditions. The ciliates survived in closed tubes within the original samples for a week, and, sometimes, even longer; thus, all investigations were performed on the specimens of the non-clonal neotype population of the original pond, taken from all of the three collected samples.

### Live observations

Live ciliates were observed for morphological details using differential interference contrast (DIC) microscopy with a Leitz (Weitzlar, Germany) microscope at a magnification of 300–1250 × with the help of a compression device [26]. For examination of the swimming behavior, ciliates were observed in a glass depression slide (3 ml) under a dissection microscope (Wild M3, Switzerland) at a magnification of 12.5–50 ×.

### Fixation and staining

Ciliates were fixed with Champy’s solution [27] and then silver nitrate-stained according to Corliss, 1953 [28]. Feulgen staining procedure after fixation in Bouin’s fluid [27] was used to reveal the nuclear apparatus.

### Cell image capturing and measurements

Computer images were captured from appropriate preparations with a digital camera (Canon PowerShot S45), automatically saved as files during optical observation at a magnification of 500–1250 ×, and used to obtain measurements of living and fixed ciliates.

Schematic line drawings were based on micrographs of typical living and impregnated cells.

### Electron microscopy

Scanning electron microscope (SEM) and transmission electron microscope (TEM) preparations were obtained as described in Modeo *et al.*[29] except for: 1. cell preservation in 2% (w/v) OsO_4_ in distilled water for SEM procedure; 2. use of 2.5% (v/v) glutaraldehyde in 0.1 M cacodylate buffer, pH 7.4, for TEM fixation.

### Fluorescence microscopy

Fluorescence microscopy to check the possible autofluorescence of cells due to the presence of methanogenic symbionts was used [30,31]. Specimens were fixed either in 4% (v/v) formaldehyde in PBS or in 2% (w/v) OsO_4_ in distilled water, and then observed at the following wavelengths: ~ 495 nm, ~ 550 nm, and UV, with both a Zeiss AxioPlan fluorescence microscope (Carl Zeiss, Oberkochen, Germany) equipped with a HBO 100W/2 mercuric vapor lamp, and a Leica DMR microscope (Leica, Switzerland) equipped with a Osram 50 W/AC L2 mercuric vapor lamp. With the latter microscope, computer images were captured from appropriate preparations by means of a dedicated software called IM1000, version 1.0.

To roughly classify ectosymbionts and possible endosymbionts harbored by *S. vorax* double fluorescence *in situ* hybridization (FISH) experiments were performed according to Ferrantini *et al.*[32]; the oligonucleotidic probes EUB338 5^′^-GCTGCCTCCCGTAGGAGT-3^′^[33], targeting most of *Eubacteria*, and Arc915R 5^′^-GTGCTCCCCCGCCAATTCCT-3^′^[34], specific for *Archaea* were used.

### 18S rRNA gene sequence obtainment

Approximately 50 organisms were individually harvested from the original sample and carefully washed three times in sterilized distilled water in order to minimize contaminations from the original medium. The washed cells were fixed in ethanol 70%. Total genomic DNA was isolated with the NucleoSpin™ Plant II DNA extraction kit (Macherey-Nagel) and stored at −20°C in aqueous solution.

A polymerase chain reaction (PCR) was performed with a Primus 96 plus thermal cycler (MWG-Biotech AG) employing the TaKaRa Ex *Taq* (TaKaRa Bio Inc.) (forward primer: 18S F9 Euk [35]; reverse primer: 18S R1513 Hypo [36]; annealing temperature: 50°C). The PCR products were sequenced in both directions using three internal primers as in Rosati *et al.*[37]. The three partially overlapping sequences were compared to each other and assembled.

### Sequence availability and phylogenetic analyses

The characterized sequence is available under the accession number [EMBL: HF547270].

The 18S rRNA gene sequence of the ciliate was aligned against those available in the SILVA 108 database [38] using the Fast Aligner algorithm of the ARB software package [39]. The alignment was then manually edited in order to optimize base-paring in the predicted rRNA stem regions. For phylogenetic analyses, gaps were coded as a fifth character, while missing data were discarded. Columns containing only one non-gap character were also discarded. The final character matrix contained 40 sequences (29 from the class Plagiopylea and 11 from the class Prostomatea as outgroup) and 1345 columns. The evolutionary model that fits best the data was selected according to the AIC parameter as calculated by jModelTest [40,41]. The TREE-PUZZLE [42] Likelihood Mapping function was employed in order to check the informational content of the data.

Phylogenetic analyses were performed with Maximum Likelihood (ML) and Bayesian Inference (BI) methods. The software PHYML [40] as provided by ARB was employed for ML, producing 1000 pseudoreplicates for bootstrapping. MrBayes 3.1.2 [43] was employed for BI, using three different runs with one cold and three heated chains each, running for 1,000,000 generations.

The tree topology was also compared against those obtained from modified character matrices. These were generated: (1) retaining only columns with at least one non-gap character conserved in at least 30% of the sequences (modified matrix 1); (2) additionally deleting all columns containing gaps (modified matrix 2); (3) removing the sequences of uncultured organisms (modified matrix 3).

## Results

### General morphology

**Figure 1:**
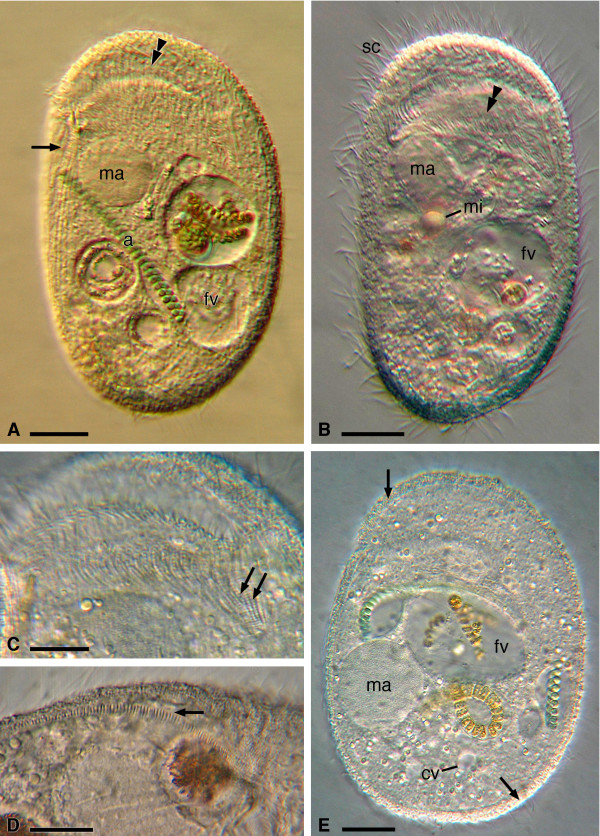
**Living observations on *Sonderia vorax*: general morphology. A**. Ventral view. Part of striated band (arrow), the macronucleus, a food vacuole, and the oral opening (double arrowhead) are visible. *S. vorax* is a algae consumer. **B**. Ventral view with focus on oral cavity (double arrowhead) and somatic ciliature. **C**. Dorsal view on the ciliate with focus on oral ciliature within the oral cavity (double arrow). **D**. Lateral view of the ciliate with emphasis on striated band (arrow). **E**. Dorsal view showing the layer of ectosymbiotic bacteria (arrow). Abbreviations: a, algae; cv, contractile vacuole; fv, food vacuole; ma, macronucleus; mi, micronucleus; sc, somatic ciliature. Scale bars = 20 μm (**A**, **B**, **E**), 10 μm (**C**, **D**).

**Figure 2:**
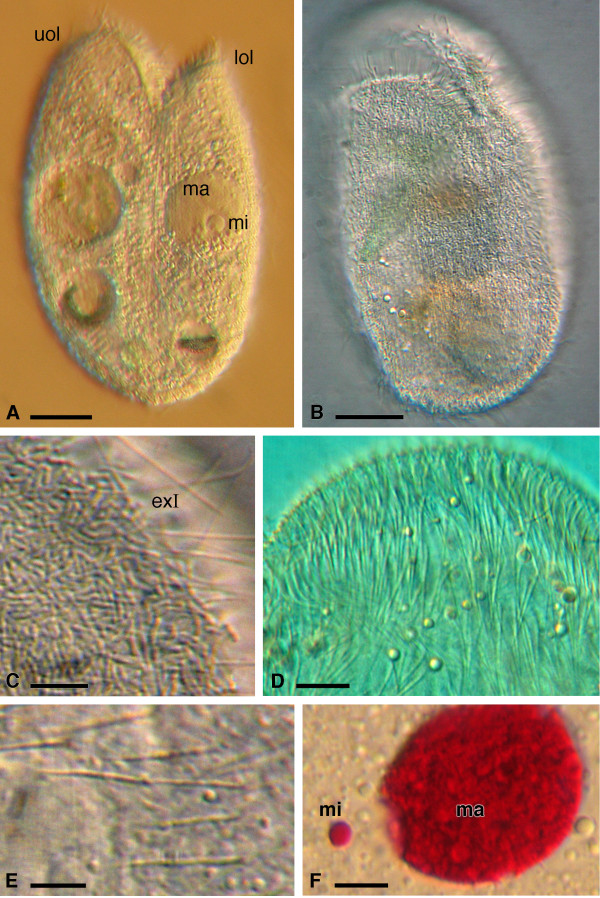
**Living observations on *Sonderia vorax*: some morphological characters. A**. Cell view from the right side of oral opening. **B**. Cell view from the left side of oral opening. **C**. Extruding longer type extrusomes. **D**. Anterior part of the cell with a number of resting longer type extrusomes. **E**. Resting longer type extrusomes at higher magnification. **F**. Nuclear apparatus after Feulgen staining. Abbreviations: exI, longer type extrusomes; lol, lower oral lip; ma, macronucleus; mi, micronucleus; uol, upper oral lip. Scale bars = 20 μm (**A**, **B**), 10 μm (**C**, **E**, **F**), 15 μm (**D**).

**Figure 3:**
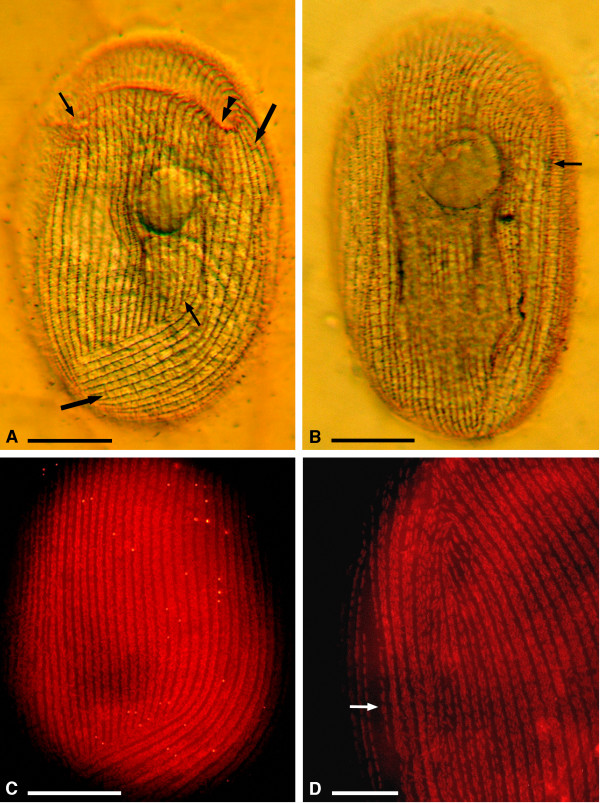
**Kinetom peculiarities of *Sonderia vorax *from silver nitrate impregnated specimens (A, B) and under fluorescent microscopy after FISH reaction (C, D). A**. Ventral kinetom consists of two parts: the central ventro-frontal part, where kineties interrupt posteriorly (arrows), and the left ventro-lateral part consisting of continuous kineties (larger arrows). The border between the first and the second part at the left side of oral opening is indicated by the double arrowhead. **B**. Dorso-lateral kinetom. The striated band (arrow) reaches the last third of ciliate body. **C**. Ventral view of a cell after FISH (probe EUB338), highlighting the covering of ectosymbionts. **D**. Right part of ciliate ventro-lateral surface after FISH (probe EUB338) with numerous ectosymbiotic bacteria distributed along kineties. Arrow points at striated band uncovered by ectosymbiotic bacteria. Scale bars = 25 μm (**A**--**C**), 10 μm (**D**).

**Figure 4:**
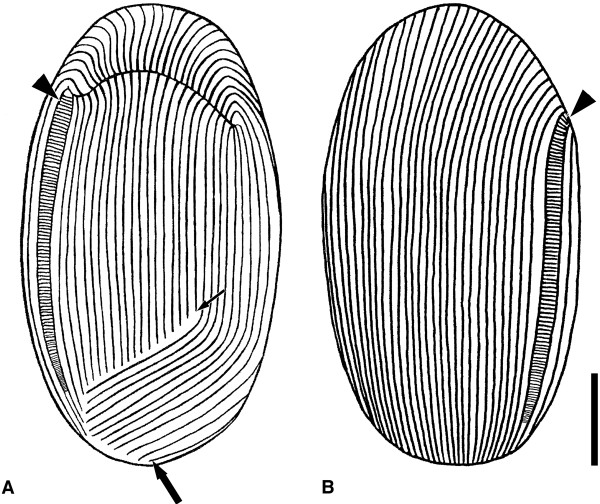
**Schematic line drawing showing the kinetom of *Sonderia vorax *according to living and silver nitrate impregnated cells. A**. Ventral view. **B**. Dorsal view. The ventro-frontal (larger arrow) and the left ventro-lateral kineties (arrow) fields as well as the striated band (arrowhead) are indicated. Scale bar = 20 μm.

**Figure 5:**
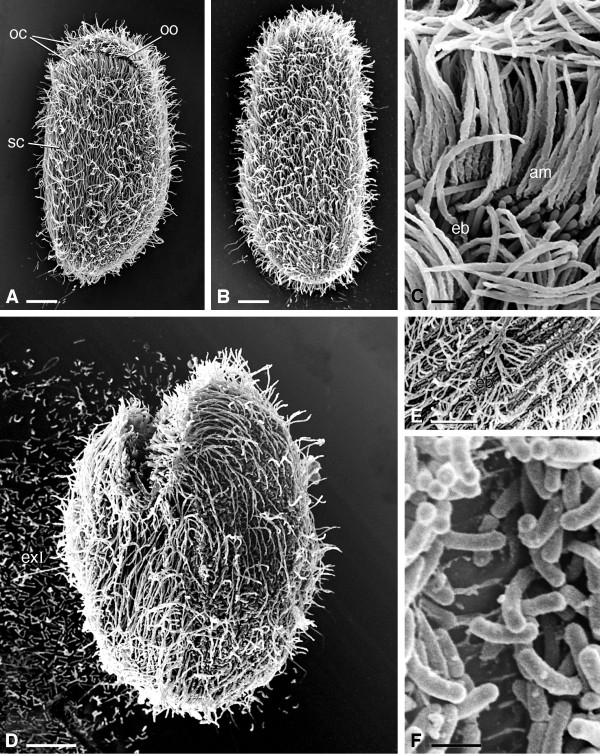
**SEM pictures of *Sonderia vorax*. A**, **B**, **D**, General morphology. **C**, **E**, **F**. Particulars. **A**. Ventral view. The oral ciliature arises from and is in continuity with the somatic ciliature. **B**. Dorsal view. **C**. On the ventral cell side, the densely packed single ciliary rows with a membranelle-like appearance at higher magnification. Ectosymbiotic bacteria cover cell surface. **D**. Lateral view of a specimen with putative ejected longer type extrusomes. **E**. Detail of the surface showing the arrangement of ectosymbiotic bacteria in parallel rows between kineties. **F**. The striated band not covered by bacteria at higher magnification. The ridge-like lamellae are visible. Abbreviations: am, ciliary rows with a membranelle-like appearance; eb, ectosymbiotic bacteria; exI, longer type extrusomes; oc, oral ciliature; oo, oral opening; sc, somatic ciliature. Scale bars = 10 μm (**A**, **B**, **D**, **E**), 1 μm (**C**, **F**).

Cells are ovoid-ellipsoid in shape with anterior and posterior ends almost equally curved (Figures 1, 2B, 3A, 3B, 4, 5B, 5D). The body is dorsoventrally flattened. *In vivo* dimensions are ~ 100–150 × 50–75 μm (130 × 69 μm on average); dimensions after fixation in Champy’s solution are ~ 80–130 × 45–70 μm (~ 113 × 65 μm on average) (Table 1). After SEM treatment, cell dimensions are ~ 89 × 42 μm on average (Figures 5A, 5B). The cell surface is uniformly ciliated with 45–62 somatic ciliary rows (~ 54 on average). On the dorsal side 25–31 rows (~ 28 on average) run parallel to each other extending to the posterior end of cell (Figures 3B, 4B, 5B). The dorso-lateral striated band at the right cell margin is visible with DIC microscope as well as on impregnated specimens, arising near the right side of the oral cavity cleft and terminating near the posterior end of the cell (Figures 3B, 4). This structure, that can be considered as a border between dorsal and ventral sides (Figures 1A, 1D, 3A, 3B, 3D, 4), is ~ 2 μm high at SEM, with ~ 0.5 μm-spaced out, ridge-like lamellae ~ 0.15 μm in thickness each (Figure 5F). A single contractile vacuole, apparently without collecting canals, is located in the posterior part of cell and opens on the dorsal side (Figure 1E); its pore was not clearly impregnated with silver staining procedure.

**Figure 6:**
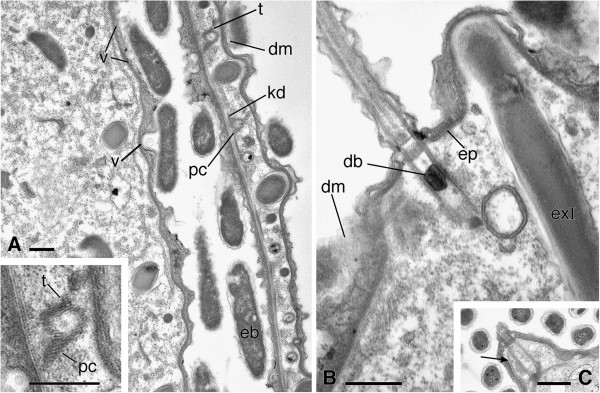
**TEM pictures of *Sonderia vorax*: cell surface, cortex, and somatic ciliature. A**. Ectosymbiotic bacteria cover the cell surface. Vesicles inside small cortical depressions are found in correspondence with ectosymbiotic bacteria. Slightly dense material is occasionally observed between ectosymbiotic bacteria and ciliate surface. Roots of monokinetids are visible: the kinetodesmal fibril, the postciliary microtubules, and the transverse microtubules. **A Inset**. Enlargment of a monokinetid root. **B**. The thick, dense epiplasm under the cortex; a dense body is visible inside somatic monokinetids. **C**. Bundles of subcortical microtubules (arrow). Abbreviations: db, dense body; dm, dense material; eb, ectosymbiotic bacteria; ep, epiplasm; exI, longer type extrusomes; kf, kinetodesmal fibril; pc, postciliary microtubules; t, transverse microtubules; v, vesicles. Scale bars = 0.5 μm.

**Figure 7:**
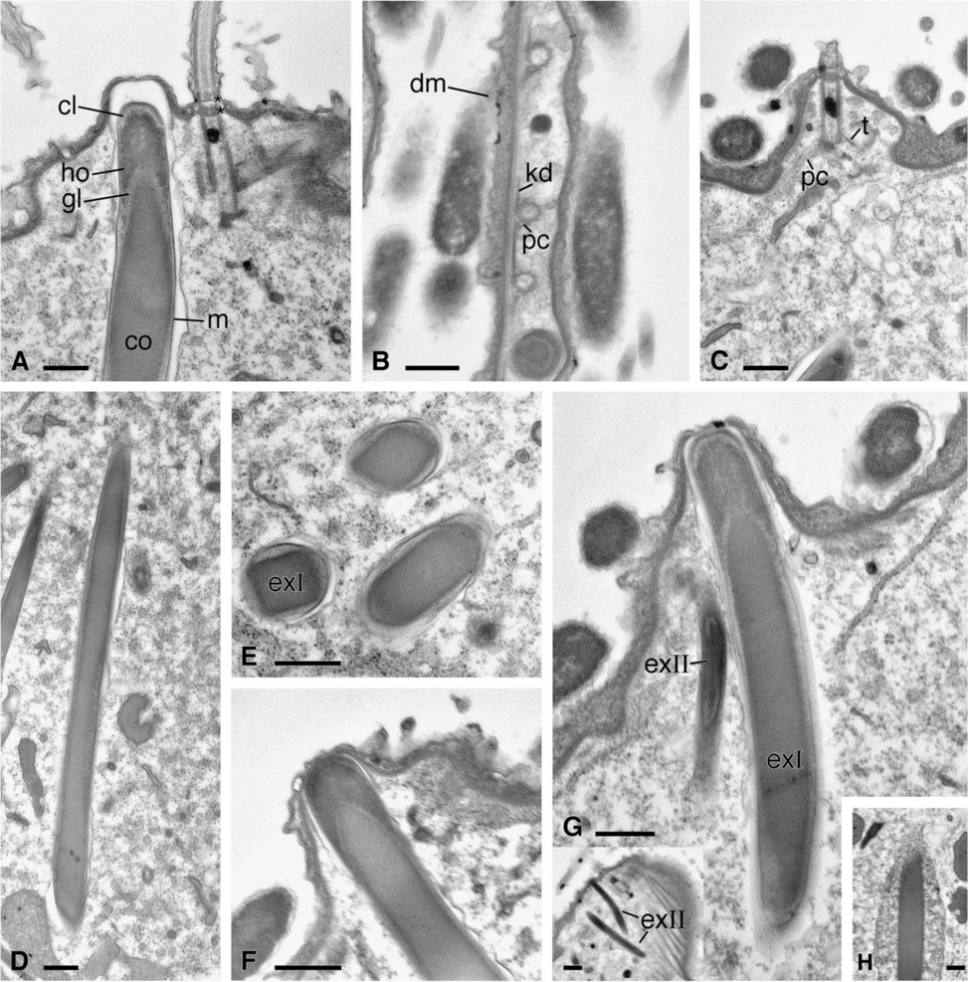
**TEM pictures of *Sonderia vorax*: somatic ciliature and extrusomes. A**. Monokinetids (in longitudinal section) with two serial terminal plates (asterisks) alternate with longer type extrusomes. These show a layered inner structure in longitudinal section (also visible in **D**, **F**--**H**). According to pictures, longer type extrusomes in five different stages are visible: developing (**H**), not yet docked (**D**), almost docked (**A**), resting (**G**), about extruding (**F**). **B**, **C**. The somatic monokinetids in cross (**B**) and longitudinal (**C**) section and part of their fibrillar associates: the kinetodesmal fibril and the postciliary microtubules; a slightly dense material is present between cortex and ectosimbionts **E**. Longer type extrusomes in cross and oblique section. **G**, **G Inset**. A second type extrusome in longitudinal section near a longer type extrusome. Abbreviations: cl, clear layer; co, core; dm, dense material; exI, longer type extrusome; exII, second type extrusome; kd, kinetodesmal fibril; gl, glanular layer; ho, hood; m, extrusome membrane; pc, postciliary microtubules. Scale bars = 0.5 μm.

**Figure 8:**
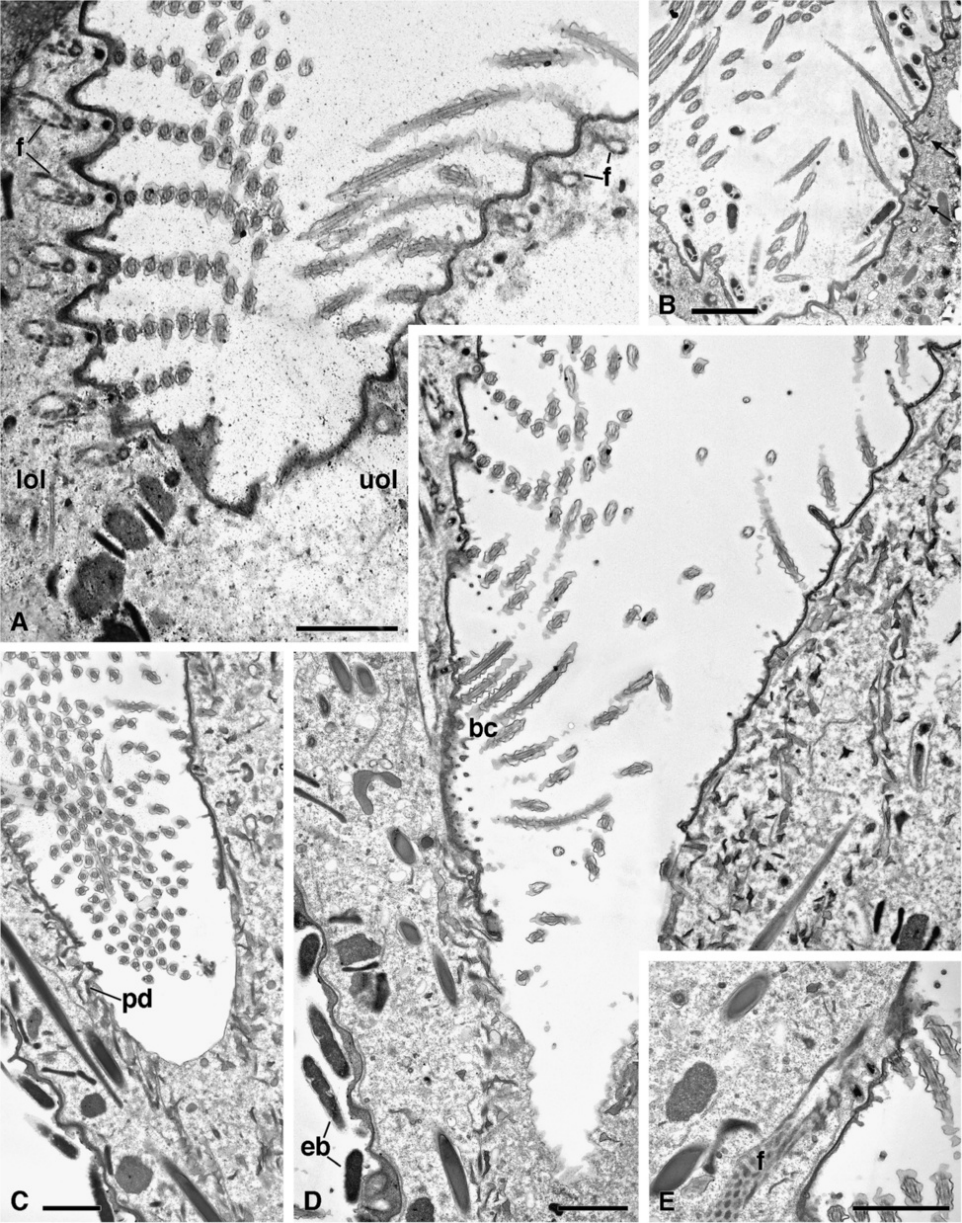
**TEM pictures of *Sonderia vorax***: **oral ciliature. A**. Cross section of adoral membranelles. The kineties of the lower oral lip are perpendicularly inserted with respect to those of the upper oral lip. The complex system of fibers interconnecting the adjacent oral kineties and the kinetosomes within each kinety is also visible in **E**. **B**. Two cilia of a kinetiy of the upper oral lip connected at the basal bodies (arrows). **C**. Section at the very end of cytostome where the cortex is lacking. **D**. Composite micrograph obtained using Adobe Photoshop 7.0 (Adobe Systems, San Jose, CA, USA). Photomontage of two pictures to show the almost complete structure of oral opening and the presence in the deep oral zone of a bundle of cilia arising perpendicularly oriented with respect to the membranelle-like ciliary rows on the lower lip and extending towards the cytostomal region. **E**. Detail of the complex system of fibers interconnecting oral kineties. Abbreviations: bc, bundle of cilia; f, fibers; lol, lower oral lip; pd, pharyngeal discs; uol, upper oral lip. Scale bars = 2 μm.

**Figure 9:**
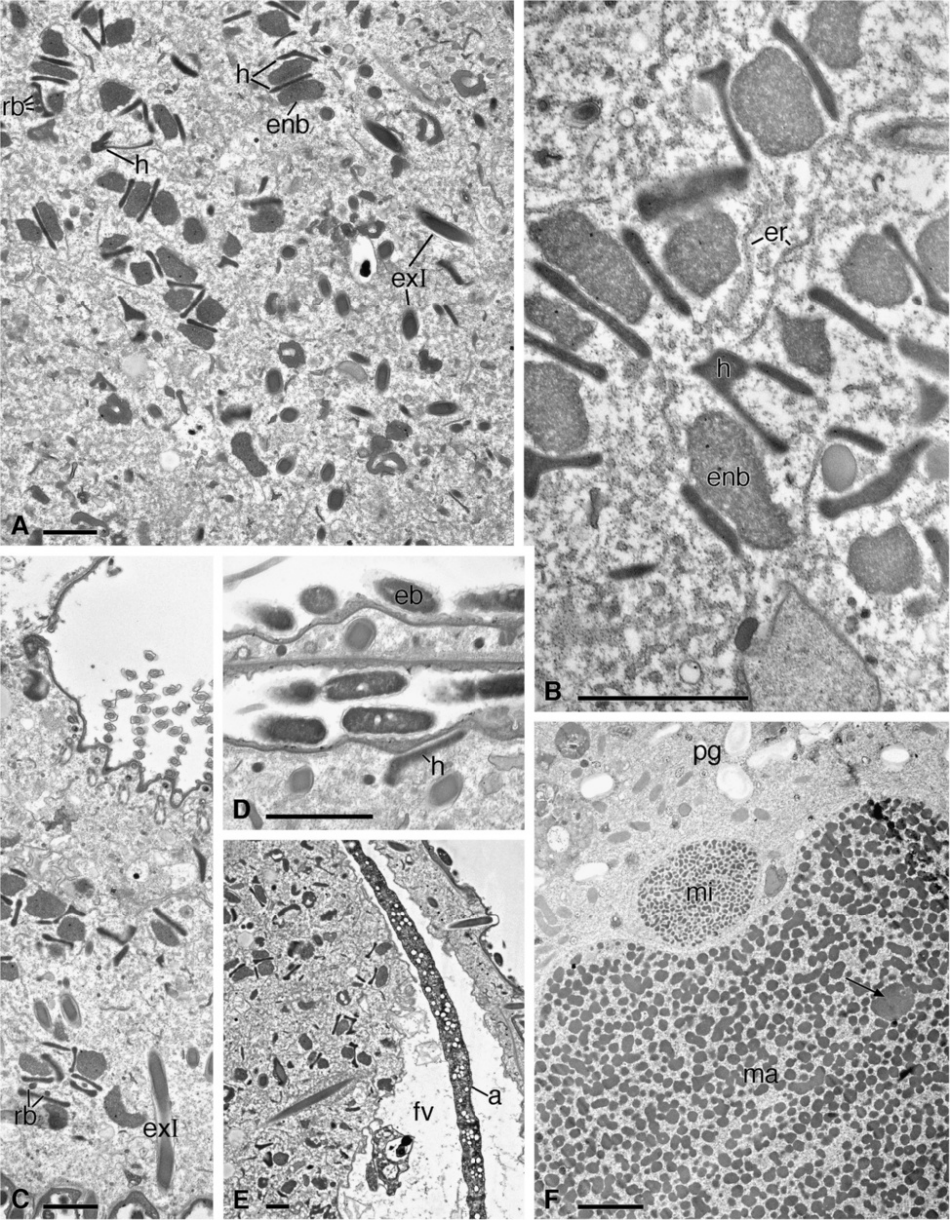
**TEM pictures of *Sonderia vorax*: cytoplasm and nuclear apparatus. A**. Various arrangements of hydrogenosomes-endosymbiotic bacteria. A few rod-shaped bacteria are visible in contact with hydrogenosomes. **B**. Various arrangements at a higher magnification; some association with the endoplasmic reticulum is visible. **C**. Rod-shaped bacteria in contact with hydrogenosomes and an obliquely cut longer type extrusome are visible. **D**. Hydrogenosomes not associated with endosymbiotic bacteria are sometimes visible strictly under the cortex, in clear correspondence with ectosymbiotic bacteria. **E**. A large food vacuole containing algae. **F**. Large paraglycogen granules, a portion of the macronucleus, and the micronucleus are visible; arrows point to a nucleolus. Abbreviations; a, algae; enb, endosymbiotic bacteria; er, endoplasmic reticulum; exI, longer type extrusome; fv, food vacuole; h, hydrogenosomes; ma, macronucleus; mi, micronucleus; pg, paraglycogen granules; rb, rod-shaped bacteria. Scale bars = 2 μm.

**Figure 10:**
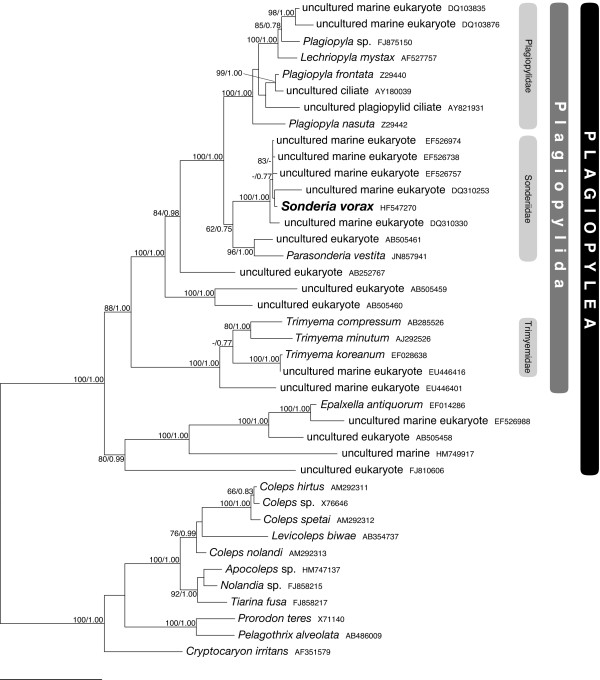
**Maximum Likelihood phylogenetic tree of the class Plagiopylea based on 18S rRNA gene sequences (unmodified character matrix, see test). **The GTR + I + G model of substitution (with the continuous gamma function approximated by four discrete categories) was employed. 92.4% of 100,000 randomly chosen quartets had well-defined topologies. Average base frequencies calculated by TREE-PUZZLE were 28.3% (A), 18.5% (C), 26.0% (G), 27.2% (T); no sequence deviated from these values (p>> 0.05). The numbers associated to each node represent bootstrap values and posterior probability, respectively (values below 60/0.75 are not shown). The bar stands for an estimated sequence divergence of 10%.

**Table 1:** **Morphometric characterization of *Sonderia vorax *Kahl, 1928**

**Characteristics**	**Min**	**Max**	**Mean**	**SD**	**CV**	**n**
Body, length	80	130	113.1	12.5	11.0	12
Body, width	45	70	64.9	5.4	8.3	12
Somatic ciliary rows (dorsal), number	25	31	28.3	2.6	9.2	17
Somatic ciliary rows (ventro-frontal), number	12	18	17.1	2.4	14.0	14
Somatic ciliary rows (ventro-lateral), number	8	13	11.4	1.8	15.8	11
Somatic ciliary rows (general), number	45	62	54.0	4.13	7.75	20
Prebuccal kineties (upper oral lip), number	25	30	27.6	2.05	7.4	3
Postbuccal kineties (lower oral lip), number	18	20	19.0	1.0	5.3	3
Macronucleus, length	25	35	32.5	3.7	11.4	15
Macronucleus, width	22	34	27.0	2.7	10.0	15
Micronucleus, number	1	1	1	0	0	20
Micronucleus, diameter	5.0	6.0	5.4	0.3	5.5	20

All measurements are in μm. Data obtained from silver impregnated or Feulgen stained (nuclear apparatus) specimens. CV, Coefficient of variation in %; Max, maximum; Mean, arithmetic mean; Min, minimum; n, number of specimens investigated; SD, standard deviation.

On the ventral side, the oral cavity opening is subapically located as a cleft orientated perpendicularly to the main body axis (Figures 1A, 1B, 3A, 5A). The oral ciliature arises from and is in continuity with the somatic ciliature (Figures 5A, 5C). It runs on the dorsal side (upper oral lip) at first perpendicularly to the front of oral cleft; then, it deviates under some angle to the left reaching the oral cavity’s deepest point (Figures 1A, 1C). The oral ciliature of the lower oral lip consists of kineties perpendicularly inserted with respect to the upper oral lip kineties and forms single ciliary rows with a membranelle-like appearance at SEM (distance between two ciliary rows: ~ 0.3 μm; length of cilia: ~ 5 μm) (Figure 5C). The oral ciliature is represented by 25–30 prebuccal (on the upper oral lip) and 18–20 postbuccal (on the lower oral lip) densely packed kineties. The depth of oral cavity is always not more than 1/3 of body length. On the ventral side, the ciliate kinetom (20–31 ciliary rows, ~ 28 on average) consists of two distinct parts: the ventro-lateral kineties (8–13 ciliary rows, ~ 11 on average), which are continuous along the cell body, and the ventro-frontal kineties (12–18 ciliary rows, ~ 17 on average), which are not continuous (Figure 4A); they start after the membranelle-like ciliary rows and end posteriorly where they meet the left ventro-lateral kineties, forming the so-called ventral secant system [9]; 4–5 rows of the latter group start from the left margin of oral cleft (Figures 3A, 4A).

Many ~ 20 μm long, slightly curved, needle-shaped extrusomes are present in the cortex (Figures 2C-E). They are mainly distributed around the oral cavity opening, but can be found in any part of the cortex and in the cytoplasm. During ejection they appear as long filaments (length at SEM: ~ 17 μm) (Figure 5D). A single quite large micronucleus (diam: 5.4 μm on average) of the compact type is situated nearby or inside the depression of the almost spherical macronucleus (27 × 32.5 μm on average) (Figure 2F). The cell surface is covered by a layer of slightly curved, rod-shaped ectosymbiotic bacteria (size at SEM: ~ 1.5-3.0 × 0.3-0.5 μm), arranged in parallel rows along interkinetal spaces (interkinetal space thickness at SEM: ~ 2.3 μm) (Figures 1E, 3C, 3D, 5C, 5E) except for striated band (Figures 3D, 5F). At SEM observation no gelatinous or mucous coating between the layer of bacteria and ciliate surface was detected (Figure 5).

### Notes of behavior

Specimens of *Sonderia vorax* rotate on the main body axis always anticlockwise (i. e. cells are left spiral swimmers). This species inhabits brackish water sites with oxygen deficiency (oxygen level 1–7%) and is mainly a consumer of diatoms and other algae.

### TEM observation

#### Cell surface and cortex

The surface of *Sonderia* is furrowed by deep longitudinal depressions separated by sharp ridges. Two rows of the rod-shaped ectosymbiotic bacteria (size: ~ 1.5–4.3 × 0.3–0.8 μm) lay in each furrow mostly with their long axis parallel to the cell surface. Two membranes delimit their dense, uniform cytoplasm that, only rarely, contains white spots. The outermost membrane is wavy and shows protruding small vesicles. In some spots it is in direct contact with host cell membrane; in some cases the irregular outline of the bacteria appears to be perfectly accommodated by irregularities in the host membrane. Where bacteria are present, the ciliate cortex forms small depressions in which vesicles are often visible (Figure 6A). Occasionally, a very thin layer of slightly dense material is barely visible between bacteria and ciliate surface (Figures 6A, 6B, 7B).

In the cortex the plasma membrane and the outer alveolar membrane are strictly associated and appear to originate from the well-developed, irregular, alveolar space. In the latter a homogeneous, dense material is always present. The inner alveolar membrane is underlined by a 40–50 nm thick dense layer (epiplasm) (Figure 6B). Longitudinal bundles of subcortical microtubules are present along the surface of the cortical ridges (Figure 6C).

#### Somatic ciliature

The kineties are composed of monokinetids inserted at the top of the cortical ridges. Kinetosomes are relatively large and ~ 1 μm long. They display a terminal plate and a secondary terminal plate in apparent continuity with the epiplasm, and often contain a dense body (Figures 6B, 7A). The somatic monokinetids have typical fibrillar associates (Figures 6A, 7A-C): a kinetodesmal fibril extending to overlap the kinetodesmal fibrils of anteriormost monokinetids; the postciliary ribbon; a short transverse ribbon originating at the opposite side of the kinetosome with respect to postciliary microtubules.

#### Extrusomes

Numerous, prominent extrusomes are distributed between the kineties. They are very long, slightly curved rods, ~ 0.6 μm in diameter. The longest longitudinal section we obtained (Figure 7D) is 9.7 μm but, as revealed by *in vivo/*SEM observation, they are certainly longer. Although the distal region of these extrusomes is differently organized with respect to the main part of the organelle, a distinct “tip” is not present (Figures 7A, 7E, 7F). Internally to their membrane an electrondense sheet covers a thin, granular layer of variable density that, in turn, surrounds a continuous dense core in which we were not able to evidence a periodicity. In cross sections this core shows a somehow squared section (Figure 7E); it maintains the same size (~ 0.5 μm) for the whole length of the organelle except at the distal region level where it is pointed and enveloped by the granular layer (Figure 7F). Then, a material with apparently the same electrondensity of the core forms a sort of hood. At this level three different layers are present and clearly evident in sections. Starting from the membrane they are: the hood, the granular layer, and a thin portion of the core (Figures 7A, 7F, 7G). When the extrusomes are positioned right beneath the plasma membrane in docking sites between the alveoli, a clear layer forms the distal end of the structure (Figure 7G). The extrusomes originate deeply in the cytoplasm. A stage of this extrusome development is shown in Figure 7H.

A second, different kind of extrusome, smaller (~ 2 × 0.2 μm) and less differentiated than that described above, is also visible (Figure 7G inset). Unfortunately in TEM preparations we never observed any type of extrusome ejected or during ejection process.

#### Oral zone ciliature

The ciliature of the upper oral lip is continuous with the somatic kineties, but the cilia are inserted in less pronounced cortical ridges perpendicularly to the body long axis (Figure 8A). They are connected at the basal bodies (Figure 8B). The kineties of the ciliature of the lower oral lip are perpendicularly inserted with respect to those of the upper lip. They form densely packed single ciliary rows with a membranelle-like appearance (Figure 8A). The kineties of the oral lips do not have kinetodesmal fibrils nor postciliary and transverse microtubules. Notwithstanding their different organization, in both the upper and the lower oral lips a complex system of fibres interconnects the adjacent oral kineties and the kinetosomes within each kinety (Figures 8A, 8E). Deeply in the oral zone, a bundle of cilia arises on the lower lip, perpendicularly oriented with respect to the membranelle-like ciliary rows but connected with them by the same complex fiber system. It extends towards the cytostomal region, i.e. where the cortex is interrupted and the zone delimited by the simple plasma membrane begins (Figure 8D). Pharyngeal disks-like structures are present in the cytoplasm surrounding this zone (Figures 8C, 8D).

#### Cytoplasm

The cytoplasm is rich in ribosomes; cisternae and tubules of endoplasmic reticulum are abundant. Well formed or developing extrusomes can be found in different cytoplasmic regions. Various hydrogenosomes-endosymbiotic bacteria assemblages, where partners alternate for position, can be observed throughout the cytoplasm in the neighbourhood of endoplasmic reticulum elements (Figures 9A-C, 9E). On the base of previous papers showing the presence of similar associations in relative genera (see Discussion), we recognized hydrogenosomes as very electron dense, double membrane bounded organelles, with a granular matrix and an irregular shape; sometimes they appeared more similar to rods (dimensions: ~ 2.0 × 0.2 μm), sometimes they appeared as flattened disks (Figures 9A, 9B). No enfoldings of the inner membrane were detected within hydrogenosomes. The endosymbiotic bacteria (size: ~ 2.0 × 0.6 μm) are not enclosed by a ciliate-derived membrane and are often irregularly shaped. Both hydrogenosomes and bacteria, but more often the latter, are found at the ends of assemblages (Figures 9A-C, 9E); the assemblages sometimes appear to be somehow in intimate association with endoplasmic reticulum (Figure 9). Occasionally a smaller, denser, rod-shaped type of bacteria (width: ~ 0.1-0.2 μm) is visible in contact with hydrogenosomes (Figures 9B, 9C). Only in a few cases hydrogenosomes not associated with endosymbionts are observed strictly under the cortex, near to the ectosymbiotic bacteria (Figure 9D).

Very large food vacuoles, containing a variety of ingested material, occupy most of the internal cytoplasm (Figure 9E). Polysaccharide reserve substances are in the form of large paraglycogen granules (Figure 9F); lipid droplets are also present.

#### Nuclei

In stationary phase, macronuclear chromatin forms small condensed bodies, in which numerous, conspicuous nucleoli are dispersed. The micronuclear chromatin is organized in a dense meshwork of branched bodies, thinner than those in the macronucleus. The chromatin occupies most of the nuclear centre and is separated from the nuclear envelope by a narrow rim of karyolimph (Figure 9F).

### Fluorescence microscopy observation

Cells observed after treatments do not autofluoresce (data not shown). Neither endo- nor ectosymbiotic bacteria are labeled by archeal specific probe Arc915R (data not shown). Ectosymbionts are marked by the universal eubacterial probe EUB338 (Figures 3C, 3D): since they cover most of the cell surface, it is not possible to discriminate signals arising from endosymbionts possibly labelled by the same probe.

### Phylogenetic analysis

The ML tree is shown in Figure 10. With one significant exception discussed below, the topology of the ingroup is almost identical in all trees calculated.

Inside class Plagiopylea, the clade containing *Epalxella antiquorum* (Odontostomatida, Epalxellidae) and four related environmental sequences is the sister group of a major cluster containing all the taxa of the order Plagiopylida. These are distributed in the Trimyemidae clade with three morphospecies of the genus *Trimyema*, the Plagiopylidae clade including the genera *Plagiopyla* (non monophyletic) and *Lechriopyla,* and the Sonderiidae clade with the sequences of *S. vorax* and *Parasonderia vestita*. All the aforementioned clades also include environmental sequences from freshwater and marine environments, either suboxic or anoxic. The five sequences most closely related to that of *S. vorax* were obtained from the supersulfidic and anoxic Framvaren Fjord (Norway).

The monophyly of the families Plagiopylidae and Trimyemidae is well supported. The status of family Sonderiidae is more dubious. It appears monophyletic when trees are calculated either on the unmodified character matrix or the modified matrix 1, although with low statistical support (62/0.75 and 60/0.75 respectively); the support is higher (97/1.00) when sequences from uncultured organisms are discarded (modified matrix 3). In trees calculated on the modified matrix 2, the *P. vestita* sequence and the closely related environmental sequences AB505461 cluster with Plagiopylidae instead, again with low support (72/0.76).

## Discussion

### Identification of our population as *Sonderia vorax* Kahl, 1928 and comparison with related species

Up to now 10 species of *Sonderia* have been described, most of them by Kahl (8 species) [1,11]. Four of the originally described *Sonderia* species were transferred to three different genera [15]: *Oncosonderia* (*Sonderia tubigula*); *Parasonderia* (*Sonderia cyclostoma* and *Sonderia kahli*); *Kahlisonderia* (*Sonderia mura*). Currently the genus consists of seven species, but the species composition is definitely different with respect to that originally proposed by Kahl. An additional species, *Sonderia vestita* (*Parasonderia vestita* according to Xu *et al.*[16]), still has an uncertain position. The main features of that ciliate do not resemble those of *Parasonderia kahli*, designated as the type species for its genus [14,15], so that Jankowski [15] proposed to keep it as *S. vestita* following Kahl [11]. Finally, the new species *Sonderia paralabiata*[44] has never been properly described; the only distinctive feature provided by the authors designates the arrangement of kinetids in groups of 3–5 kinetosomes. This is in contradiction with the later statement of Lynn [12] that “the somatic kinetids are monokinetids in the sonderiids, plagiopylids, and trimyemids”. Nevertheless, 3 other sonderiids – *S. vestita, Sonderia labiata, S. paralabiata,* and maybe *Sonderia sinuata*, with di- and even up to pentakinetids in the somatic ciliature have been reported [4,5,16,44].

Most of the descriptions of *Sonderia* spp. did not include silver impregnation and other staining methods, and were based upon only a few morphological markers, which sometimes could not provide easy discrimination between the ciliates (Table 2). Xu *et al.*[16] stated that “the oral structure […] is one of the most important diagnostic characters of genera within the family Sonderiidae”, but these data are not yet available for the majority of *Sonderia* species. Nevertheless our *Sonderia* fits well the original description and pictures of *S. vorax* in the publications of Kahl [1,11]. However, this species has never been reinvestigated with modern analytical tools after the original isolation from marshes in Oldesloe (1928) and in the Island of Sylt (1931). Thus, some of the distinctive characteristics of the ciliate which were not mentioned in the original description [1] are lacking (see Table 2).

**Table 2:** **Comparison between the morphology and morphometry of *Sonderia vorax *and other similar-sized species of *Sonderia *according to selected literature data**^**a**^

**Character**	***Sonderia pharyngea *Kirby, 1934**	***Sonderia labiata *Fauré-Fremiet & Tuffrau, 1955**	***Sonderia sinuata *Kahl, 1931**	***Sonderia sinuata *Borror, 1972**	***Sonderia sinuata *Dragesco & Dragesco-Kernéis, 1986**	***Sonderia sinuata *Sola *et al., *1989**	***Sonderia sinuata *Al-Rasheid, 2001**	***Sonderia vorax *Kahl, 1928**	***Sonderia vorax *Khal, 1931**	***Sonderia vorax *present study**
cell length, μm	84--110 (IV?)	160--180 (IV?)	240--250 (IV?)	145--164	180--240 (IV?)	132--176 (OF)	90--120 (IV?)	60--150 (IV)	70--180 (IV)	~ 100--150 (IV)
cell width, μm	48--65 (IV?)	~60--75^c^ (IV?)	nd	81--113	nd	78.1--105.6 (OF)	82--95 (IV?)	~ 30--75^b^ (IV?)	~ 30--75^c^ (IV?)	~ 50--75 (IV)
differentiation of ventral kinetom	–	–†	+†	nd	–†	+	–†	+†	+†	+
n of ventro-frontal kineties	nd	nd	nd	nd	nd	~ 30	nd	nd	nd	12--18
n of ventro-lateral kineties	nd	nd	nd	nd	nd	~ 8†	nd	nd	nd	8--13
n of ventral kineties (total)	nd	35--40	nd	nd	24	~ 38†	nd	nd	nd	20--31
n of dorsal kineties	nd	35--40	nd	nd	20--30	45	nd	nd	nd	25--31
composition of somatic kineties	nd	dk or tk	nd	nd	dk	mk; dk within the vestibular cavity	nd	nd	nd	mk
n and composition of oral kineties	nd	23† prebuccal + 17† postbuccal; nd	nd	nd	nd	30† prebuccal + 20† postbuccal; dk	nd	nd	nd	25-30 prebuccal + 18–20 postbuccal; mk and dk
peristome size : cell size ratio	> 1/2 (IV)	~ 1/2†	~ 1/2	~ 1/2	~ 1/2	~ 1/2	~ 1/2	~ 1/2 †	< 1/2†	~ 1/3
dsb size: cell size ratio	nd	~ 1/2†	1	1	nd	1	nd	nd	nd	+ ~1
ma, length, μm	15--20 (IV)	~ 35† (SI?)	nd	nd	~ 25† (PS)	20.9--33.0 (OF)	25	~ 20†	nd	27 (average)(SI)
ma, width, μm	15--20 (IV)	~ 35† (SI?)	nd	nd	~ 25† (PS)	22.0--30.8 (OF)	25	~ 20†	nd	32.5 (average) (SI)
mi, number	1	nd	1	1	1	1	1	1	1	1
mi, diameter, μm	nd	nd	nd	nd	nd	nd	4--7	nd	nd	~ 5.4 (average)
exI, size, μm	7--9 (IV)	~20 (SI?)	nd	nd	22--26	nd	6--10	nd	~ 20	~ 20 (IV)
exI, amount and distribution	few; sparse and unevenly distributed throughout the cell	many, inserted in the cortex	nd	nd	nd	nd	many, unevenly distributed throughout the cell†	many; inserted closely to surface †	many; inserted closely to surface †	many; inserted in the cortex and sometimes free in the cytoplasm
swimming rotation	nd	nd	nd	nd	nd	nd	nd	nd	nd	anticlockwise
habitat (salinity)	hypersaline water (35--100 ‰)	brackish water (1--6 ‰)	brackish water (3--20 ‰)	seawater	brackish water	fresh water	sea water (35--38 ‰)	brackish water (5--20 ‰)	brackish water (5--20 ‰)	brackish water (4--8 ‰)

^a^, In Kahl [11] 7 species of *Sonderia *were included; in Carey [76] 8 *Sonderia *spp. were mentioned, but the majority of them were even more poorly described than those included in this table; ^b^, original indication was “ratio lengh : width 2 : 1”; ^c^, original indication was “ratio lengh : width 2½ : 1”; +, character present; –, character absent; dsb, dorso-laterally striated band; exI, longer type extrusomes; IV, *in vivo*; ma, macronucleus; mk, monokinetids; mi, micronucleus; n, number; nd, character not mentioned in the reference; †, character derived from pictures/drawings; OF, osmium tetroxide fixation; PS, protargol staining; SI, silver impregnation; tk, trikinetids.

*S. vorax* can be easily separated from some of the similar-sized species, *S. pharyngea* and *S. labiata*, because these ciliates do not show a differentiation of ventral kinetom, the so-called secant system [9] (Table 2). On the contrary, this feature is probably shared between *S. vorax* and *S. sinuata*[9], although it has not been indicated neither by Dragesco & Dragesco-Kernéis [4] nor by Al-Rasheid [45]. Anyway, *S. vorax* differs from *S. sinuata* for: cell size (180–240 μm *vs.* 100–150 μm); buccal cavity size (1/2 of cell body *vs.* 1/3 of cell body); number of dorsal kineties (45 *vs.* 25–31); size (and probably structure) of the micronucleus [4,9] (Table 2). The oral ciliature consists of monokinetids in *S. vorax* while some contradictions do exist in the literature for *S. sinuata*[4,9].

### General remarks on SEM and TEM analyses

Papers dealing with SEM and TEM observation on the complete cell structure of order Plagiopylida are respectively lacking and scarce. Fine structure descriptions of representatives of family Sonderiidae are limited to the data on *S. vorax* and *Sonderia* sp. reported by Fenchel *et al.*[46], in a study on the interaction between those ciliates and other marine organisms (the so-called “sulphide fauna”) and their prokaryote symbionts. As general information about the determination of their species are lacking, on the basis of the few TEM pictures and data supplied, we could just state the congenerity of our species with *S. vorax* studied by those authors, but nothing could be argued concerning their conspecificity. Beside the comparison with TEM data reported by Fenchel *et al.*[46], we also took the opportunity to shed light on general ultrastructure of Plagiopylida; thus, we made a larger comparison with available data on plagiopylid genera such as *Lechriopyla* and *Plagiopyla*[30,47-53], as well as on the single trimyemid genus *Trimyema*[19,23,51,54,55].

### SEM observation and ectosymbionts

Ectosymbiotic bacteria somewhat covering the cell surface of ciliates have been widely reported (e.g. [56]). Those borne by the hypotrich *Euplotidium* spp., referred to as epixenosomes [57,58], are peculiar extrusive symbionts nearly identical at the ultrastructural level to the spherical episymbiotic bacteria of the euglenozoan *Bihospites bacati*[59], which lives in oxygen-poor habitats. The latter species also bears rod-shaped ectosymbiotic bacteria; these appear to be widespread in protists living in oxygen-poor habitats, as they have been also described in both flagellates such as *Calkinsia aureus*[60] and *Postgaardi mariagerensis*[61] and ciliates such as *Parablepharisma* spp., *Metopus* spp., and *Sonderia* spp. [6,8,11,46,62,63].

In previous papers on *Sonderia* spp. the presence of a gelatinous coat between the ectosymbiotic bacteria and the ciliate plasma membrane was either reported as clearly visible under light microscope [5,9] or at least supposed [46]. Although ectosymbiotic bacteria covering our species appeared partly aggregated on the slide when detached from the ciliate (Figure 5D), a gelatinous coat between ectosymbiotic bacteria and plasma membrane was not evidenced by SEM, while by TEM only a slightly dense layer of material in a few occasions was observed (see below). This result, an apparent discrepancy between SEM and TEM observation, is also evident in pictures of other papers dealing with protists living in oxygen-poor habitats that bear ectosymbionts underlined by a glycocalyx on their cell surface [59,60].

Curiously, the presence of ectosymbionts as a common feature of the Sonderiidae was not even mentioned in two of the most solid recent ciliate reviews [12,13]. However, this could be in our opinion a good morpho-biological feature to discriminate members of Sonderiidae family. The ectosymbionts of *S. vorax* were marked by the universal eubacterial probe in FISH experiments; this indicates their affiliation to *Eubacteria*.

The height of the striated band on the right surface measured at SEM in *S. vorax* fits that reported by Lynn [12] in the general description of somatic structures of the families Plagiopylidae and Sonderiidae; thus, we confirmed the size of this structure in sonderiids, but the meaning of this peculiar cortex feature still remains unknown.

### TEM observations and endosymbionts

#### Cell surface and cortex

The bacteria covering the surface of *Sonderia* spp. described by Fenchel *et al.*[46] are 1.5-2.5 × 0.35-0.40 μm and are visible within the oral vestibulum but not in the ciliate peristome; the ectosymbiotic bacteria covering the surface of our *S. vorax* share with them this kind of localization but appear slightly longer and wider, and more tidily oriented.

As in other previously described Plagiopylida [47,54], the somatic cortex of *S. vorax* includes a homogeneous, dense alveolar material, of variable thickness. In our opinion, this material could have been interpreted, under the light microscope, as the gelatinous coat reported by previous authors [5,9,46]. Alternatively, it is possible that the coat could be formed, under particular conditions, by this alveolar material released by means of the vesicles visible inside the small depressions of the cortex of our *S. vorax* where bacteria are localized.

#### Somatic ciliature

Kineties of *S. vorax* are composed of monokinetids as is typical of Plagiopylea [12] with the possible exception of *Parasonderia vestita* according to Xu *et al.*[16]. Somatic cilia at the tops of cortical ridges in *S. vorax* appear typical of Plagiopylidae in contrast to kinetosome arrangement between the ridges observed in Trimyemidae [12].

Our observations on the kinetid pattern of *S. vorax* fit the interpretation of Lynn [12]: in Plagiopylida the transverse ribbon has a radial orientation on the opposite side with respect to postciliary microtubules and a very short trajectory.

Kinetosomes are longer then those described in *Lechriopyla mystax* and *Plagiopyla minuta* by Berger and Lynn [47] (~ 1.0 μm *vs.* 0.65 μm) but those two organisms share with *S. vorax* the presence of two terminal plates. The presence of dense material inside somatic and oral kinetosomes of *S. vorax* was also reported by Detcheva *et al.*[54] in *Trimyema compressum*.

#### Extrusomes

The longer extrusomes of our species can be considered a novel kind of extrusive organelle so far unknown [64,65]. Actually, their complex structure does not fit the definition of mucocysts reported for the class Plagiopylea by Lynn [12], neither if the latter are meant as “elongate and rod-shaped mucocysts” as described for plagiopylids and sonderiids, nor if they are meant as “spheroidal mucoysts” of trimyemids. In particular, the organelles, although similarly distributed (between kinetids of the kineties), appear different in shape and layer organization from extrusomes described by Berger & Lynn [47]. Neither our longer extrusomes, nor those described by Berger & Lynn [47], nor the longer extrusomes reported by de Puytorac *et al.*[53] in *Plagiopyla nasuta* can be assimilated with classical trichocysts: none of them actually exhibit the typical trichocyst organization in a distal, distinct tip and a larger striated basal portion [64,65]. Unfortunately, a comparison with the “large trichocysts” reported in *Sonderia* sp. (and *Plagiopyla frontata*) by Fenchel *et al.*[46] is unfeasible because the authors only evidenced them in a picture without any comment; however, those organelles share with our extrusomes at least the layered inner structure.

Only de Puytorac *et al.*[53] reported the presence of two kinds of extrusomes in a representative of plagiopylids, i.e. *P. nasuta*: a first curved type already cited and a smaller, not fully described second type; both of them were, in the authors’ opinion, very different from mucocysts. Comparison is not possible between the second type of extrusome we observed by TEM in *S. vorax* and that of *P. nasuta* due to the lack of fine structure details. However, due to their small sizes and low abundance, these organelles could likely be overlooked during former descriptions of *Sonderia* spp. [5,9,46].

#### Oral zone ciliature

The oral kinetosomes of our *S. vorax* appear typical of Plagiopylida [12].

#### Hydrogenosomes and endosymbionts

As expected on the basis of previous ultrastructural studies concerning free-living as well as endocommensal Plagiopylida [23,30,31,46-52,54,55,66], we observed the absence of mitochondria and the presence of hydrogenosomes in *S. vorax*. Hydrogenosomes are descendents of mitochondria that anaerobically oxidize pyruvate to acetate and CO_2_, producing molecular hydrogen and ATP [67-70]; the organelles present in our species should reasonably cover the same functions.

In sulphide ciliates (among which there were two species of *Sonderia*) Fenchel *et al.*[46] described organelles called “microbodies”, later identified as hydrogenosomes by Finlay & Fenchel [30]. The similarity in the general aspect between the hydrogenosomes we found in our species and that of *Sonderia* spp. previously studied is restricted to electron density, double membrane bounding, irregular shape, granular matrix, and close contact with endoplasmic reticulum. The sizes, especially for the rod-shaped hydrogenosomes herein described, and the localization reported by Fenchel *et al.*[46] appear actually very different. Moreover, hydrogenosomes of different *Sonderia* spp. were not associated with endosymbiotic bacteria. This feature, not so far highlighted in *Sonderia* spp. [30,46], seems to be on the contrary widespread in Plagiopylida: besides our *S. vorax*, it was reported in *Plagiopyla minuta* and *Lechriopyla mystax*[47,48]; the latter species showed associations resembling those observed in *P. frontata*[51]. Furthermore, *P. nasuta* was recently restudied after successful cultivation [52], and was found to harbour two types of endosymbiotic bacteria: one of them, a methanogen, was almost always observed in very close association with hydrogenosomes. These “peculiar packets” appeared different to those observed in *P. frontata*; indeed, according to molecular analysis, they are different methanogens [19].

The endobacteria-hydrogenosomes associations we observed in our *S. vorax* resemble those described in *P. minuta* and *L. mystax* by Berger & Lynn [48] and those reported in *P. frontata* by Finlay & Fenchel [31] in their morphology and their frequent association with endoplasmic reticulum. Nevertheless, at variance with the bacteria described in the above mentioned papers and by Lynn [12] for the whole order Plagiopylida, the endobacteria we observed do not autofluoresce. Thus, they appear not to be methanogens. Although their identity cannot be directly established from our data, the negative results with the archeal probe in FISH experiments suggest that they probably belong to *Eubacteria*. This result is also in line with the observation of Fenchel & Finlay [50] who already reported the presence in genus *Sonderia* of non-methanogenic endosymbionts as notable exception among free-living ciliates bearing hydrogenosomes. Associations between hydrogenosomes and non-methanogenic bacteria in anaerobic ciliates were also reported by Finlay *et al.*[71] from a sulphide-rich solution lake in Spain, and by Clarke *et al.*[72] in the scuticociliate *Cyclidium porcatum*, which contains organized complexes of three different components: hydrogenosomes, methanogenic bacteria, and non-methanogenic bacteria.

To conclude this comparison of fine structure details within order Plagiopylida, in *Trimyema* spp. stable methanogenic endosymbiotic bacteria-hydrogenosomes associations were reported [23,55]. Finlay *et al.*[23] found that endosymbionts tend to change morphology aiming at maximizing the contact with hydrogenosomes. This feature was not observed in the non-methanogenic endosymbionts of our *S. vorax*, but several hydrogenosomes show irregular forms which tend to “embrace” endobacteria as to reach maximum contact with them.

Our TEM data are the first to reveal the presence in Sonderiidae of close associations between hydrogenosomes and endosymbiotic bacteria; thus, this feature can actually be considered typical of order Plagiopylida supporting Lynn [12]. Nevertheless, the endosymbionts involved in the arrangements are not methanogens. Of course it could be questionable whether our cells could have lost methanogenic endosymbiotic bacteria during their permanence in laboratory: for example, in *Trimyema* sp. rod-shaped methanogens were easily lost during monoclonal culturing, which lasted four years [55]. In spite of several attempts, we did not succeed to cultivate monoclonal strains of our species and we performed our experiments soon after collection of samples; this allows us to be pretty confident in our findings.

Finally, our species generally tends not to harbour endosymbiotic bacteria not associated with hydrogenosomes; this is again in contrast with the paper of Fenchel *et al.*[46], where “rod-shaped”, “intracellular particles resembling bacteria” appeared mostly abundant directly beneath cell membranes of *Sonderia* spp. Nevertheless, looking at the pictures of those endosymbionts and considering only their general morphology (i.e. excluding size) they somehow resemble the bacteria we observed associated with hydrogenosomes in our species; this could mean that in the same species (or at least genus) the presence/absence of arrangements formed by hydrogenosomes and endosymbiotic bacteria is likely depending on ecological stimuli and/or constraints.

#### Nuclei

In stationary phase the macronuclear chromatin of *S. vorax* resembles that reported in some Heterotrichea (e.g. *Chattonidium setense*[29]; *Peritromus kahli*[37]). Concerning its fine structure, the micronucleus reminds of the compact type micronucleus described by Fokin [73].

### Phylogeny

Our inference partially differs from that of Xu *et al.*[16] about the position of *Parasonderia vestita*. It was considered very closely related to Plagiopylidae in their article; in most of our trees it is instead more closely related to *S. vorax* and its associated environmental sequences. Both their result and ours are poorly supported, and we have demonstrated that the position of *P. vestita* can change according to how the character matrix is built. Thus, we prefer to leave the question of the Sonderiidae monophyly open, until more data will be obtained and a reliable topology could be inferred. The monophyly of genus *Plagiopyla* is not recovered, because the sequence of *Lechriopyla mystax* is nested inside those of the former genus. In none of the papers providing sequences of Plagiopylidae representatives a detailed morphological analysis of the studied organisms is supplied as well. Thus, the possibility that some of them were simply misidentified cannot be excluded. However, a more likely explanation is that the classical morphological characters employed to distinguish between these taxa are not good indicators of evolutionary relatedness, because either plesiomorphic or too vague. It is important to notice, however, that the morphologically similar Plagiopylidae and Sonderiidae do form a robust clade in molecular phylogeny: this clade is clearly separated from Trimyemidae, which members possess a distinctively different morphology.

There are many environmental sequences available in the databases that clearly cluster with characterized plagiopylean species. It is remarkable that all these sequences come from anoxic or suboxic, and often sulfidic, environments. *S. vorax*-like sequences were apparently obtained only from the Framvaren Fjord (North Sea, Norway) in two different studies [74,75]. Interestingly, the North Sea is also one of the places where Kahl originally collected ciliates of the genus *Sonderia*.

## Conclusions

In the present paper we redescribed and neotypified the plagiopylid ciliate *Sonderia vorax* from a brackish water pond along the Italian coastlines. By means of the applied multidisciplinary analytical approach, more familiarity with the typical features of this poorly-known ciliate was gained. Moreover, in the light of the comparison between our findings and the scarce available literature on the order Plagiopylida, some previous systematics interpretations concerning this taxon were confirmed and some difficulties and ambiguities in the classification became as well evident. Our data significantly contribute to the general understanding of the overall diversity of the Plagiopylida. Nevertheless, it still remains a largely unexplored ciliate order: multidisciplinary analytical studies from a larger number of representatives are needed to clarify the phylogenetic relationships within this group.

## Diagnosis

### *Sonderia vorax* Kahl, 1928

1928 *Sonderia vorax* – Kahl, *Archiv Hydrobiol***19:**93**–**95, Figs 20a-c. [1]

1931 *Sonderia vorax* – Kahl, *Tierwelt Dtl***21:**269, Fig. 14. [11]

1934 *Sonderia vorax* – Kirby, *Archiv Protistenknd***82:**116. [7]

1969 *Sonderia vorax* – Fenchel, *Ophelia***6:**1**–**182. [6]

1972 *Sonderia vorax* – Borror, *Acta Protozool***10:**29**–**71. [2]

1992 S*onderia vorax* – Carey, *Marine Interstitial Ciliates* 103–104, Fig. 359. [76]

### Diagnosis of neotype material

Body dimensions *in vivo*: ~ 130 × 69 μm (on average). Outline body shape ovoid-ellipsoid with rounded ends, flattened up. On average 56 ciliary rows with ventral kinetom differentiated into two parts (ventral secant system): the ventro-frontal part (~ 17 rows on average) which breaks off posteriorly where it meets from the left the continuous ventro-lateral part (~ 11 rows on average at the ventral surface left). Kineties composed of monokinetids placed at the top of cortical ridges. Depth of oral cavity never more than 1/3 of body length. Oral ciliature arising in continuity with somatic ciliature; oral ciliature of the lower oral lip (18–20 postbuccal rows) consists of kineties perpendicularly inserted with respect to the upper oral lip kineties (25–30 prebuccal rows) and forms single ciliary membranelle-like rows. Dorso-laterally striated band arising near the right side of oral cavity and terminating near cell posterior end. A number of long needle-shaped extrusomes in body cortex visible under light microscope; a second smaller type visible only by TEM. A single contractile vacuole in the posterior part of the dorsal side of the cell. One quite large micronucleus of the “compact” type situated in the depression of the nearly spherical macronucleus. Different hydrogenosomes-endosymbiotic bacteria assemblages distributed throughout the cytoplasm, often in the neighbourhood of endoplasmic reticulum elements. Cell surface completely covered except for striated band by a layer of slightly curved, rod-shaped ectosymbiotic bacteria, arranged in parallel rows along interkinetal spaces. The ciliate rotates about main body axis always anticlockwise (left spiral swimming) and inhabits brackish water sites with oxygen deficiency (level 1-7%).

### Neotypification and neotype material

No useable type material (type or voucher slides) is available so far from any of *Sonderia vorax* populations [15,17]. The original description [1] is incomplete and apparently based on living observations only. Thus, it seems wise to define *S. vorax* by the designation of a neotype [77,78]. Validation of the neotype according to Article 75.3 of the ICZN [77] is justified by the following particulars: (i) the systematic status of *S. vorax* (it was considered as valid species after Kahl [1], but the description has never been improved according to a modern set of morphological methods); (ii) the differences between *S. vorax* and related taxa (see Discussion and Table 2); (iii) the neotype specimens (Figures 3A, 3B) representing neotype population from the Ligurian coastline pond (Pisa district, Tuscany, Italy) are described in details (see above); thus, recognition of the neotype designated is ensured; (iv) it is generally known that no type material is available from species described by Kahl; (v) there is strong evidence that the neotype is consistent with *S. vorax* as originally described by Kahl [1]; (vi) however, the neotype does not come from a site very near to the original type locality (Oldesloe salt marshes, Hamburg region, Germany). Neotype population of the ciliate was found in the middle part of Ligurian Sea (coastline pond nearby Serchio river mouth, Tuscany, Italy), roughly distance: ~ 1000 km; however, both sites are brackish water. Most ciliates, especially marine ones, are cosmopolitans [79], hence this point should not be over-interpreted. A detailed description of the new type locality, that is the sample site of the neotype population, is given in Material and Methods; (vii). One neotype slide of silver nitrate-impregnated specimens (slide № S-11), collected from the pond in Ligurian coastline, Pisa district, Tuscany, Italy, (sampling date 05 October 2005; collector S. I. Fokin), one permanent Feulgen staining preparation (slide № S-17), and Epon-embedded material for TEM investigation have been deposited in the collection of the Museo di Storia Naturale e del Territorio dell’Università di Pisa, Calci (PI), Italy. Two further neotype slides of silver nitrate-impregnated specimens (slides № S-12 and S-14) have been deposited in the slide collection of the Laboratory of Invertebrate Zoology, Biological Research Institute, St. Petersburg State University, St. Petersburg, Russia.

### Neotype locality

Owing to the neotypification, the sampling site of the neotype population is the new (valid) type locality of *Sonderia vorax*: brackish water pond along the coastline of Ligurian Sea close to Serchio River mouth (Pisa district, Tuscany, Italy; 43°47^′^16″N, 10°16^′^02″E).

### Etymology

The derivation of the species-group name is in the original description by Kahl [1].

### Gene sequence

The 18S rRNA gene sequence of *S. vorax* is available under the accession number [EMBL: HF547270].

## Abbreviations

SEM: Scanning electron microscope/Microscopy; TEM: Transmission electron microscope/Microscopy; FISH: Fluorescence *in situ *hybridization; PCR: Polymerase chain reaction.

## Authors’ contribution

LM and GR performed SEM and TEM analyses and the comparative research on the related literature; SIF performed *in vivo*, silver-staining and FISH analyses, and reviewed the species identification problem; IA and FF obtained the 18S rRNA gene sequence; VB performed the phylogenetic analyses; FV and GP supervised the work and provided advices on the draft. All authors read and approved the final manuscript.
